# Dental Economics

**Published:** 1885-07

**Authors:** A. Berry


### Dental Economics.

BY A. BERRY D.D.S.

Read before the Mad River Dental Society.

GENTLEMEN OF THE MAD RIVER VALLEY DENTAL SOCIETY:—

I propose to advance a few ideas on dental economics in accordance with one of Webster's definitions of economy: "a judicious application of time, of labor, and the instruments of labor."

In the dental office every thing should be kept in order, with all instruments and appliances ready for use; that no time be lost in preparation when a patient calls. Appointments should be met promptly, and operations performed without unnecessary delay. It is well to procure gold foil by the ounce, and other materials for filling teeth in considerable quantities, to avoid waste of time in obtaining them in smaller amounts when wanted.

For extracting teeth the most complete set of forceps of best forms and sizes, as well as a dozen or more elevators of skillfully devised shapes, should be at command.

If an important instrument becomes unfit for use, it should be repaired at once, or replaced by another. If the band of the dental engine fails, and another can not be obtained readily, a common chalk line affords a substitute as good in every respect as the original.

If nitrous oxide is used and made at the office, it is economical to have the gasometer not less than one hundred and twenty gallons capacity, as it saves time and trouble to make gas oftener with a gasometer of usual size.

In the laboratory there should be the best appointments for obtaining good results with the least expenditure of time. The tools, lathes, furnace, and vulcanizers, should be in excellent condition.

Newark plaster of paris of good quality is prudently procured by the barrel. To have it set quickly and make a hard cast it should be worked rapidly for a considerable period before pouring.

Several hundred dollars worth of teeth kept on hand will prove a paying investment, obviating the necessity of getting them from dealers in emergencies, as well as being advantageous to select from when the patient is present. There should be a good assortment of the various sizes, forms, and colors. Those similar in color should be put together to facilitate selections.

Gold and other materials for mounting teeth should be ready for any contingency.

The Buckeye vulcanizer (invented by Dr. C. H. James of this society), secured by one screw, without numerous or complicated fastenings, simple in construction and easily manipulated, is preferable to any other, but it should be more strongly made and of little larger diameter than those usually in the market.

A pin in the side of its rim so set that it will rest in a notch in the top when in place, keeping it constantly in the same position, is very convenient. Asbestos packing is much more durable than rubber.

Flasks held together by wedges possess decided advantages over those with bolts.

For testing the degree of heat of a vulcanizer a thermometer is unnecessary. A few drops of water applied will, after a little attention to the process, prove less troublesome and quicker than scrutinizing a thermometer for testing the heat, and with equally satisfactory results. And besides, it is economical to keep free of the occasional and needless expense of one dollar for one thermometer, or three dollars for a dozen.

A vulcanizer should never be used without a safety valve, guarding against the fear and danger of explosion; of which there is little risk with prudent management. If a fire in stove or grate is at hand, putting the vulcanizer in a kettle and raising the temperature to the boiling point, before placing it over the heat for vulcanizing, saves time and gas. Above 112 degrees the heat is best applied steadily so as to raise it slowly in about eighty minutes to 310 degrees, and after holding it there thirty minutes the heat may be stopped, and the vulcanizer left in place until cold. Rubber hardened in this manner is much stronger than that vulcanized in the usual time and at a higher temperature.

